# Evidence for a Shared Mechanism in the Formation of Urea-Induced Kinetic and Equilibrium Intermediates of Horse Apomyoglobin from Ultrarapid Mixing Experiments

**DOI:** 10.1371/journal.pone.0134238

**Published:** 2015-08-05

**Authors:** Takuya Mizukami, Yukiko Abe, Kosuke Maki

**Affiliations:** Graduate School of Science, Nagoya University, Nagoya, Aichi, Japan; Universidade Nova de Lisboa, PORTUGAL

## Abstract

In this study, the equivalence of the kinetic mechanisms of the formation of urea-induced kinetic folding intermediates and non-native equilibrium states was investigated in apomyoglobin. Despite having similar structural properties, equilibrium and kinetic intermediates accumulate under different conditions and via different mechanisms, and it remains unknown whether their formation involves shared or distinct kinetic mechanisms. To investigate the potential mechanisms of formation, the refolding and unfolding kinetics of horse apomyoglobin were measured by continuous- and stopped-flow fluorescence over a time range from approximately 100 μs to 10 s, along with equilibrium unfolding transitions, as a function of urea concentration at pH 6.0 and 8°C. The formation of a kinetic intermediate was observed over a wider range of urea concentrations (0–2.2 M) than the formation of the native state (0–1.6 M). Additionally, the kinetic intermediate remained populated as the predominant equilibrium state under conditions where the native and unfolded states were unstable (at ~0.7–2 M urea). A continuous shift from the kinetic to the equilibrium intermediate was observed as urea concentrations increased from 0 M to ~2 M, which indicates that these states share a common kinetic folding mechanism. This finding supports the conclusion that these intermediates are equivalent. Our results in turn suggest that the regions of the protein that resist denaturant perturbations form during the earlier stages of folding, which further supports the structural equivalence of transient and equilibrium intermediates. An additional folding intermediate accumulated within ~140 μs of refolding and an unfolding intermediate accumulated in <1 ms of unfolding. Finally, by using quantitative modeling, we showed that a five-state sequential scheme appropriately describes the folding mechanism of horse apomyoglobin.

## Introduction

Kinetic intermediates are often observed during the folding of proteins with more than ~100 amino acid residues. Early kinetic intermediates are usually characterized as compact, partially folded states containing native-like secondary structures, and may even exhibit specific tertiary interactions for some side-chain pairs. Based on their native-like features, it has been suggested that kinetic intermediates represent productive states playing an important role in guiding unstructured polypeptide chains to fold into specific native structures [1,2]. A complete understanding of protein folding requires detailed characterization of the early folding intermediates, which initially eluded investigation, due to the limited time resolution of conventional kinetic techniques, such as stopped-flow (SF) methods.

Under moderately denaturing conditions, some proteins accumulate equilibrium intermediates that are structurally and thermodynamically similar to kinetic intermediates [1]. These equilibrium states have been investigated as potential analogues of short-lived kinetic intermediates in order to overcome the difficulty in characterizing the latter in detail [3–11]. This made it possible to elucidate properties of equilibrium intermediates such as topology (fold) [12] and structural cooperativity [13,14], which would be difficult to determine by kinetic experiments alone. However, these properties can be used for describing folding mechanisms only if the equilibrium states are truly equivalent to kinetic intermediates. Even if equilibrium and kinetic intermediates have similar structural properties, which has been shown for several proteins, they generally accumulate under different conditions, and may form by different kinetic mechanisms. Typically, kinetic folding intermediates accumulate transiently under strongly native conditions, whereas non-native equilibrium intermediates tend to be populated under moderately denaturing conditions, including conditions that do not favor folding, such as high salt concentrations at low pH [1,2]. Therefore, it is crucial to investigate whether the two types of intermediates share the same kinetic folding mechanism and why intermediates are often structurally similar despite differences in manner and conditions of their accumulation. Recent advances in experimental techniques, such as laser-induced temperature jump and continuous-flow (CF) methods, have extended the time resolution of kinetic measurements into the nanosecond to microsecond range [15–18]. There are, however, only a few studies in which the equivalence of transient and stable intermediates has been confirmed by directly following the kinetics of their formation.

**Fig 1:**
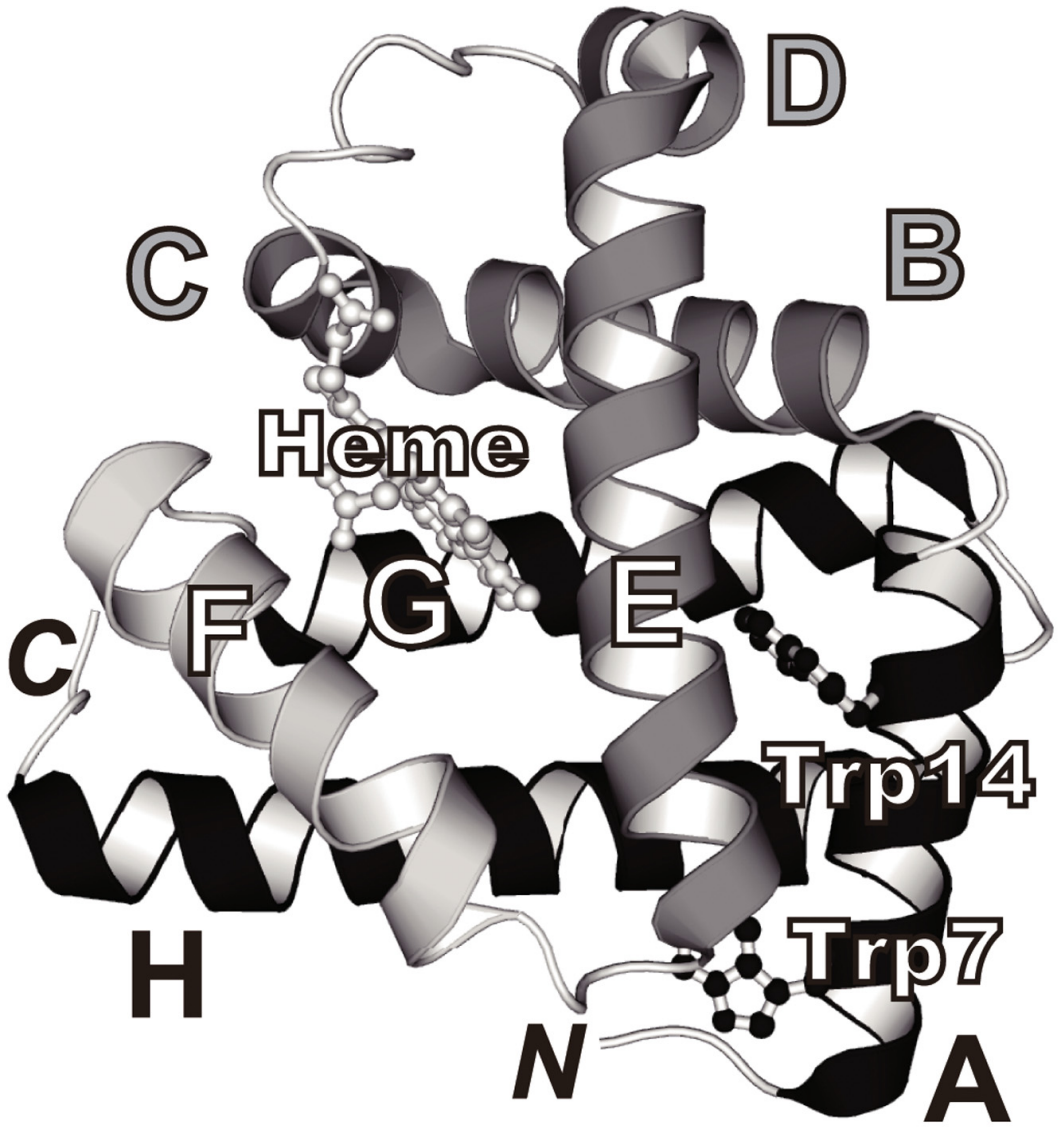
Ribbon diagram of horse skeletal muscle myoglobin on a crystallographic structure (PDB: 1AZI). The heme group (light gray) and two tryptophanyl side chains (black) are shown in the ball-and-stick model. The native state of myoglobin contains eight helices (A–H), whereas the native state of h-apoMb contains seven helices (A–E, G, and H; black and gray). The intermediate of apoMb exhibits a helical structure in the A-, G-, and H-helix regions (black). The F-helix is shown in light gray. The figure was prepared using PyMol (DeLano Scientific).

Apomyoglobin (apoMb) is an excellent model protein for investigating these questions because the properties of kinetic and equilibrium intermediates and folding mechanisms have been studied extensively [19,20]. Myoglobin is a heme protein with 153 amino acid residues consisting of eight helices (A–H) that adopt a globin fold [21]. Early studies found that the protein exhibits fully native properties without heme, but is slightly less compact and more disordered than the holo form [22–24]. ApoMb, which lacks a heme group, accumulates equilibrium unfolding intermediates under various moderately denaturing conditions [25–27], including acidic conditions in the presence of salt [28,29]. Equilibrium unfolding experiments on sperm whale apomyoglobin (sw-apoMb) indicated that the pH/urea-induced intermediate was approximated by a single thermodynamic state [30]. In addition, there was no clear transition between the salt-induced and pH-induced intermediates for horse skeletal muscle apomyoglobin (h-apoMb), which suggests that a detectable thermodynamic barrier does not exist between them [28]. Further structural characterization revealed that the intermediate at pH 4 (hereafter known as "the pH 4 intermediate") is compact with a structural core consisting of a native-like helical structure in the A-, G-, H-, and part of the B-helix regions [31–34]; however, under physiological conditions, it forms a compact, native structure similar to that of native myoglobin, with the F-helix, the N-terminus of the G-helix, and some loops unstructured (Fig 1) [35,36]. In contrast, the acid unfolded state at pH 2.3 was reported to be highly flexible with residual helical structures in the A-, H-, and D- to N-terminus of the E-helix regions in dynamic equilibrium with the unfolded state [33,37,38], which is consistent with previous studies of peptide fragments [39–41]. In another study, the addition of 8 M urea to the solution at pH 2.3 further denatured the protein with virtually no residual structures [42]. The folding kinetics of sw-apoMb was studied by pulsed hydrogen/deuterium (H/D) exchange experiments, and these revealed that helical structure is formed in the A-, G-, H-, and part of the B- helices of the native structure on a millisecond-to-second time scale during the folding reaction at pH 6.1 [6]. The intermediate ensemble has a hydrophobic core surrounded by native-like secondary structures with specific interactions between side-chains [43,44]. Further kinetic studies of folding indicated that sw-apoMb follows a sequential folding pathway [45] and accumulates at least two kinetic intermediates [46,47]. H-apoMb also folds via two kinetic intermediates with a compact size and considerable fraction of helical structure on a submillisecond-to-second time scale [48]. A submillisecond pulsed H/D exchange experiment with sw-apoMb revealed that a compact intermediate is formed with a helical structure in the A-, G-, and H-helix regions within 0.4 ms of the refolding [5]. The effects of protonation and temperature on the denaturation of apoMb and myoglobin were also investigated in previous simulation studies [49–51]. Although the solution conditions in which the equilibrium intermediate accumulates are far from native conditions, i.e., the conditions where kinetic intermediates transiently accumulate, the equilibrium and kinetic states exhibit similar structural properties (e.g., spectroscopic properties and patterns of amide protection in H/D exchange) [5–7]. If they are equivalent, the kinetic and equilibrium intermediates should share a common kinetic mechanism of formation. Although the rate-limiting step of the pH-induced folding reactions of sw-apoMb from kinetic and equilibrium intermediates was found to be similar by comparing their refolding kinetics initiated at pH 2.2, 3.4, and 4.2 [46], conventional kinetic measurements were too slow for a direct comparison of the kinetics of formation of transient and equilibrium intermediates.

Here, we aimed to address these issues by systematically investigating the refolding/unfolding kinetics and equilibrium unfolding induced by urea in h-apoMb. By using fluorescence-detected CF and SF measurements, we directly observed the formation of intermediates as kinetic phases in the refolding and unfolding reactions over a range of urea concentrations, while observing the burst phase intermediates formed within the dead-time of the CF refolding experiment. The results indicated that at least five states, including the native and unfolded states, are associated with the refolding/unfolding reactions of h-apoMb. Using quantitative modeling, the protein’s folding behavior can be fully described by a five-state sequential scheme. The results indicated that transient and equilibrium intermediates of h-apoMb are formed by a shared kinetic mechanism (although they accumulate under distinct conditions) and that the transient intermediate is converted into a non-native equilibrium state as urea concentrations increase. This study is complimentary to a recent study on the structure formation and unfolding of the intermediates of sw-apoMb at pH 4.0 [52].

## Materials and Methods

### Chemicals

Horse skeletal muscle myoglobin was purchased from Sigma (St. Louis, MO), and a specially prepared reagent grade urea for biochemical use was purchased from Nakalai Tesque Inc. (Kyoto, Japan). All other chemicals were either specially prepared or guaranteed reagent-grade chemicals. An Atago 3T refractometer was used to determine the concentration of urea with a refractive index of 589 nm. H-apoMb was prepared by removing the heme from myoglobin according to the methods of Teale and Uzawa *et al*. [48,53]. All appropriate solutions were filtered by using a membrane filter (pore size of 0.20 or 0.45 μm) before they were used in the experiments.

### Equilibrium fluorescence and circular dichroism measurements and fitting

For fluorescence and circular dichroism (CD) measurements, sample solutions were prepared at pH 6.0, 4.0, and 2.0 as follows. An acid-unfolded protein solution (in HCl at pH 2.0) was passed through a Sephadex G-25 column (PD-10; GE Healthcare) equilibrated with 12 mM sodium citrate at pH 6.0. Sample solutions at pH 6.0 and 4.0 containing various concentrations of urea (0–8.0 M) were prepared by six-fold dilution of the protein stock solution, resulting in a 12 mM sodium citrate buffer containing an appropriate concentration of urea. To prepare the sample solution at pH 2.0, the stock solution (in HCl at pH 2.0) passed through the Sephadex G-25 column was diluted with HCl at pH 2.0. The protein concentration for the fluorescence and CD measurements was 5 μM. Fluorescence emission spectra from 300 nm to 450 nm were recorded on a Jasco FP-777 spectrofluorometer (Tokyo, Japan) using an excitation wavelength of 295 nm at 8°C using circulating water. Equilibrium CD measurements were performed using a Jasco J-600S spectropolarimeter (Tokyo, Japan) with a Peltier device at 0–20°C, and the path length of the sample cuvettes was 1.0 mm. The equilibrium unfolding transition curves were fitted to a three-state model by non-linear least squares fitting [54–56] via a global fitting algorithm in IGOR software (Wavemetrics Inc., Lake Oswego OR). Helix contents were calculated by using the ellipticity values at 222 nm according to Chen *et al*., Sabelko *et al*., and Dasmeh and Kepp [51,57,58].

### Kinetic measurements and fitting

For CF measurements, the refolding reactions were initiated by two-fold dilution of the acid-unfolded protein solution (in HCl at pH 2.0) with 24 mM sodium citrate containing appropriate concentrations of urea to give a final pH of 6.0 whereas the unfolding reactions were initiated by two-fold dilution of the protein solution (in 12 mM sodium citrate at pH 6.0) with 12 mM sodium citrate (pH 6.0) containing appropriate urea concentrations. The final condition for CF measurements was 20–40 μM h-apoMb/12 mM sodium citrate/0–4.1 M urea at pH 6.0. For SF measurements, the refolding reactions were initiated by six-fold dilution of the protein solution in HCl at pH 2.0 and in 0.8 M urea/12 mM sodium citrate at pH 6.0 with refolding buffer containing 14.4 mM and 12 mM sodium citrate, respectively, and appropriate concentrations of urea. The unfolding reactions were initiated by six-fold dilution of the protein solution in 12 mM sodium citrate at pH 6.0 with unfolding buffer containing 12 mM sodium citrate and appropriate concentrations of urea at pH 6.0. The final condition for SF measurements was 5 μM h-apoMb/12 mM sodium citrate/0–7.6 M urea at pH 6.0. For measurement of refolding and unfolding, time-dependent fluorescence change was monitored using an optical cutoff filter (50% transmittance at 305 nm) with an excitation wavelength at 295 nm. All kinetic experiments were conducted at 8°C. CF measurement was performed as previously described by Shastry *et al*. [59]. SF measurement were carried out on an Applied Photophysics SX-17 stopped-flow device (Surrey, UK). The dead-times of the CF and SF devices, which were 102–175 μs and 5.3 ms respectively, were calibrated by measuring the bimolecular quenching of *N*-acetyl-L-tryptophanamide fluorescence with *N*-bromosuccinimide [60].

The kinetic traces obtained by CF and SF measurements were fitted using non-linear least squares fitting to the following equation:

$$F_{\text{obs}}\left(t\right)=F_{\text{eq}}+\sum F_{i}e^{-\lambda_{i}t}$$ (1)

where *F*_obs_ (*t*) and *F*_eq_ are the fluorescence intensities at time *t* of the reaction and at a time after the reaction has reached equilibrium, respectively, and *F*_*i*_ and *λ*_*i*_ are the amplitude and apparent rate constant of the *i*-th phase (*i* = 1 and 2 for refolding, and *i* = 3 and 2 for unfolding). The cumulative amplitudes, which give the fluorescence intensities in the absence of the preceding phases of refolding and unfolding, are defined as *F*_0R2_ = *F*_eq_ + *F*_2_ and *F*_0R1_ = *F*_eq_ + *F*_2_ + *F*_1_ for the refolding initiated at pH 2.0, and *F*_0U2_ = *F*_eq_ + *F*_2_ and *F*_0U1_ = *F*_eq_ + *F*_2_ + *F*_3_ for the unfolding (*F*_eq_ represents the fluorescence intensity at equilibrium).

### Quantitative modeling of kinetics

Standard numeric methods were applied to solve the system of linear differential equations describing a particular kinetic scheme, with IGOR software used to determine the eigenvalues and eigenvectors of the corresponding rate matrix. The kinetic parameters were manually optimized to fit the experimental data. The urea-concentration dependence of the elementary rate constants was assumed to follow Eq 2 [61]:

$$\text{ln}k_{ij}=\text{ln}k_{ij}^{0}+\frac{m_{ij}^{‡}}{RT}\left[\text{urea}\right]$$ (2)

where *k* ^0^_*ij*_ is the elementary rate constant in the absence of urea, and *m* ^‡^_*ij*_ is the corresponding slope. *R* and *T* are the gas constant and absolute temperature, respectively. Based on the associated rate matrix, the elementary rate constants and the slopes were explored systematically to model the rate constants and amplitudes obtained from the kinetic measurements and equilibrium unfolding experiments. Free energy diagrams were calculated from the elementary rate parameters obtained. The free energy of the relevant states and the transition states was calculated as a function of the α-value as the reaction coordinate at representative urea concentrations. The α-value was defined by a sum of *m* ^‡^_*ij*_ from N to a given state normalized by the sum of all *m* ^‡^_*ij*_, which is the measure of the change in solvent-accessible surface area relative to N. The activation energy for crossing the barriers between states was calculated as follows:

$$\Delta G_{ij}^{‡}=-RT\text{ln}\left(\frac{k_{ij}^{0}}{A_{0}}\right)-m_{ij}^{‡}\left[\text{urea}\right]$$ (3)

where an arbitrary value of 10^6^ s^-1^ was used for the pre-exponential factor, *A*_0_. Details of the quantitative modeling are described in S1 File (Methods in S1 File).

## Results

### Urea-induced equilibrium unfolding

**Fig 2:**
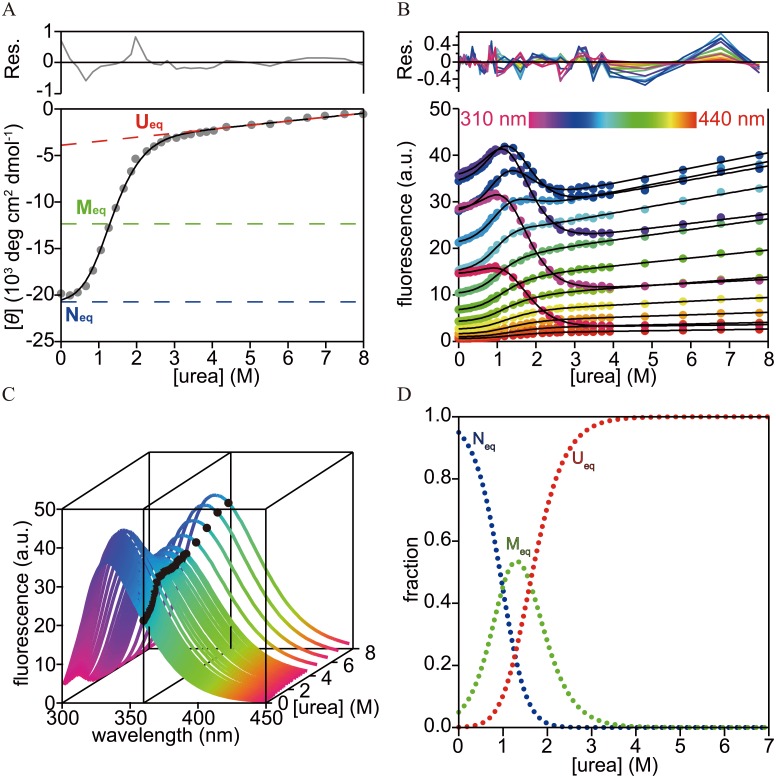
Urea-induced equilibrium unfolding of h-apoMb in 12 mM sodium citrate at pH 6.0 and 8°C. Unfolding transition curves monitored by (A) ellipticity at 222 nm and (B) the tryptophan fluorescence spectrum (310–440 nm). The solid lines are unfolding transition curves predicted by the global fitting of a three-state model (Scheme 1) along with the residuals (upper panel). (A) Broken lines are baselines of N_eq_ (blue), M_eq_ (green), and U_eq_ (red) predicted by the global fitting. (B) Unfolding curves are coded with different colors. (C) A collection of fluorescence spectra of h-apoMb as a function of urea concentration measured at pH 6.0 and 8°C. Black circles represent an unfolding transition curve monitored by the fluorescence emission at 360 nm. The transition curves in (B) were obtained by transposing data in (C). (D) The fractions of each species as a function of the urea concentration obtained by the equilibrium unfolding experiments. The fractional N_eq_, M_eq_, and U_eq_ are shown in blue, green, and red, respectively.

Fig 2 shows the urea-induced equilibrium unfolding of h-apoMb measured by monitoring the changes in ellipticity at 222 nm (Fig 2A) and the Trp fluorescence emission spectra (300–450 nm) upon excitation at 295 nm (Fig 2B and 2C). The measurements were performed in 12 mM sodium citrate at pH 6.0 and 8°C. These unfolding transition curves were similar to those previously reported for sw-apoMb [30,44]. The transition curves monitored by fluorescence at 310–360 nm exhibited two transitions: one at ~1 M urea with an enhancement and the other at ~2 M urea with a decrease. In contrast, those monitored by fluorescence at >370 nm and ellipticity at 222 nm apparently exhibited a single transition at ~1.5 M and ~1 M urea, respectively. They were fitted by non-linear least squares fitting using a global fitting algorithm [9,55,56] to an equilibrium three-state model consisting of the native (N_eq_), equilibrium intermediate (M_eq_), and unfolded (U_eq_) states as follows:

$${\text{U}}_{\text{eq}}⇌{\text{M}}_{\text{eq}}⇌{\text{N}}_{\text{eq}}$$ Scheme 1

which yielded a set of global parameters (midpoint urea concentrations of U_eq_ ↔ M_eq_ and M_eq_ ↔ N_eq_ transitions, *C*_m1_, and *C*_m2_, and the corresponding *m*-values, *m*_1_ and *m*_2_ for each transition) and local parameters (fluorescence intensities at 300–450 nm and ellipticity at 222 nm for N_eq_, M_eq_, and U_eq_ at 0 M urea, and the slope of the baselines). A linear approximation of the urea-dependence of the baseline as well as the thermodynamic stability of each species was based on the work of Pace and that of Santoro and Bolen [55,56]. The thermodynamic parameters are listed in Table 1. The fractions of the three species were also calculated as a function of urea concentration (Fig 2D). At 0 M urea, h-apoMb consisted of a mixture of N_eq_ (~95%) and M_eq_ (~5%). The fraction of M_eq_ increased from ~5% at 0 M urea, at the expense of the fraction of N_eq_, until it reached a maximum at 1.3 M urea, which was subsequently followed by a decrease with the increasing fraction of U_eq_. We also investigated the stability of h-apoMb vs. temperature to rule out possible cold denaturation [26] at 8°C, the temperature used in this study. Fig A in S1 File shows that ellipticity at 222 nm decreased with temperature down to 4°C, below which cold denaturation occurs. This confirms that h-apoMb maintained its native structure at pH 6.0 and 8°C in the absence of urea.

**Table 1:** Thermodynamic parameters for urea-induced equilibrium unfolding of hapoMb at pH 6.0 and 8°C.

	*i*	states	*m*_*i*_ (kcal/mol/M)	*C*_mi_ (M)	Δ*G* ^0^_*i*_ (kcal/mol)
**Equilibrium** ^a^	**1**	N_eq_ M_eq_	1.7 ± 0.1	1.0 ± 0.1	1.7 ± 0.1
	**2**	M_eq_ U_eq_	1.4 ± 0.1	1.6 ± 0.1	2.2 ± 0.1
	**3**	N_eq_ U_eq_	3.0 ± 0.1^b^	1.3 ± 0.1	3.8 ± 0.1^b^
**Kinetics** ^c^	**1**	N_eq_ M_eq_	1.4	0.6	0.9
	**2**	M_eq_ U_eq_	1.4	1.6	2.2
	**3**	N_eq_ U_eq_	2.8	1.1	3.1

^a^ Thermodynamic parameters obtained by fitting a collection of the equilibrium unfolding transition curves with a global fitting algorithm to a three-state model (Scheme 1). Error estimates for equilibrium parameters are based on goodness-of-fit (± one standard deviation).

^b^ Δ*G* ^0^_3_ and *m*_3_ were calculated by adding the corresponding thermodynamic parameter for the N_eq_ ↔ M_eq_ and M_eq_ ↔ U_eq_ transitions.

^c^ Thermodynamic parameters were calculated from the elementary rate constants and kinetic *m*-values (Table C in S1 File) estimated by quantitative modeling. N_eq_ is assumed to consist of N and N', M_eq_ is assumed to be equivalent to M, and U_eq_ is assumed to consist of I and U.

The tryptophan fluorescence spectra and ellipticity at 222 nm of N_eq_, M_eq_, and U_eq_ linearly extrapolated to 0 M urea were obtained as local parameters of the global fitting (Fig B in S1 File). At 0 M urea, the fluorescence spectrum and the ellipticity at 222 nm resembled those of N_eq_ derived from the global fitting, which confirms that N_eq_ is the dominant species at 0 M urea, pH 6.0, and 8°C. The ellipticity value at 222 nm estimated for N_eq_ would be slightly less than that at neutral pH because of the local destabilization of the helices [25,62]. The fluorescence spectrum and ellipticity at 222 nm of M_eq_ derived from the global fitting were similar to those of the predominant equilibrium intermediate at pH 4 (i.e., the pH 4 intermediate). Indeed, the helix contents of M_eq_, calculated using the ellipticity at 222 nm, were only ~5% lower than those of the pH 4 intermediate (Table A in S1 File). The slight reduction may have arisen due to the local destabilization of the helices or from the ambiguity in the slope of the baseline of M_eq_, which was not well defined because of the apparent single-step transition curve monitored by ellipticity. In addition, the overall structure of these h-apoMb intermediates was indicated to be similar to that of the pH 4 intermediate of sw-apoMb because 1) helix contents were similar (~0.4 for sw-apoMb [33]) and 2) amino acid sequences between h- and sw-apoMbs were highly homologous, albeit with slightly less helix propensity for h-apoMb (Table B in S1 File). This finding is supported by a previous molecular dynamics simulation study of the unfolding of sperm whale and horse myoglobin [51], which indicated an overall structural similarity of the intermediates transiently accumulated in thermal unfolding (although some difference from the apo-proteins was observed in the unfolding of the D/E-helix regions due to the presence of heme). The fluorescence spectrum and ellipticity of U_eq_ were more similar to those at pH 2.0 than to those at ~8 M urea and pH 6.0. Indeed, the helix contents estimated by the ellipticity values of U_eq_ were almost identical to those at pH 2.0 at 222 nm, with only 1% difference observed (Table A in S1 File). They were also consistent with those of the acid unfolded state of sw-apoMb at pH 2.3 (~0.1 for sw-apoMb [33]). These results provide further indication that the overall residual structure of U_eq_ is similar to that of the acid unfolded state of sw-apoMb. In contrast, almost no residual secondary structure was expected for the unfolded state at ~8 M urea. It should be noted that the differences in fluorescence intensity and CD properties between U_eq_ and ~8 M urea were expected from the slope of the baselines in the unfolding region.

### Urea-concentration dependence of the refolding and unfolding kinetics

**Fig 3:**
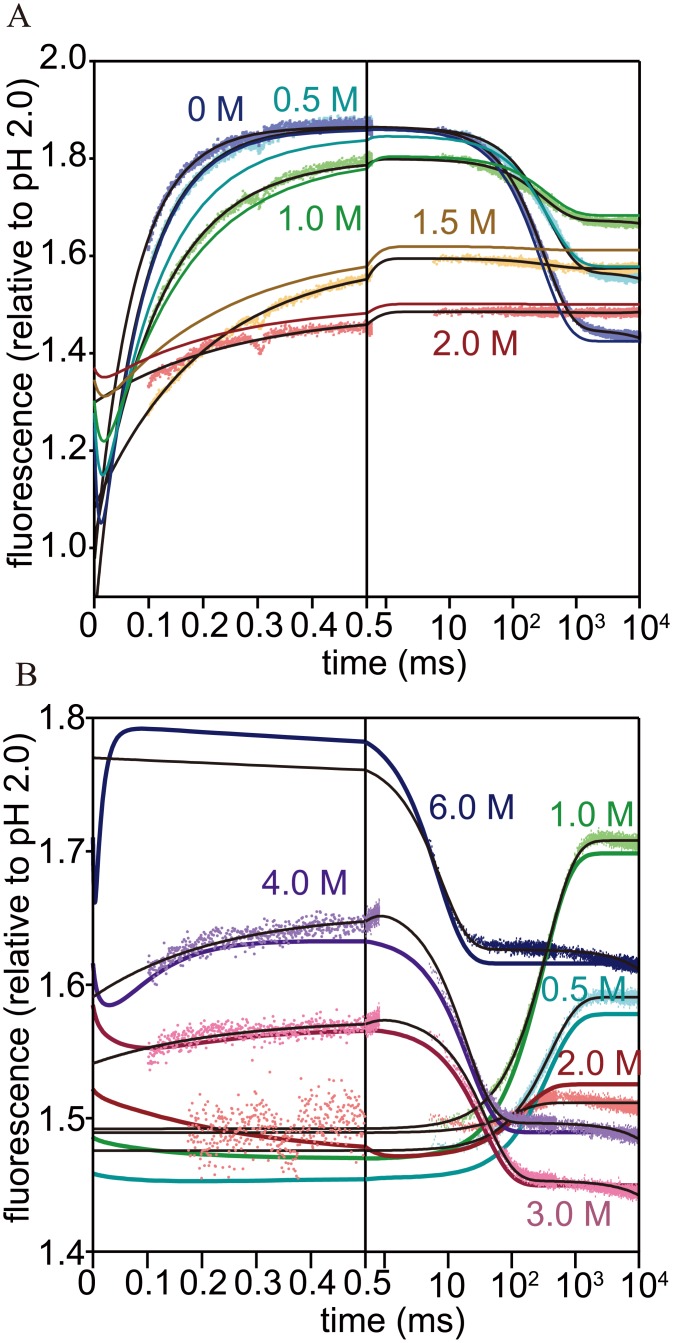
The kinetic traces of refolding and unfolding reactions of h-apoMb. The kinetic traces of (A) refolding and (B) unfolding reactions were measured by CF and SF fluorescence methods at various urea concentrations, pH 6.0, and 8°C. The fluorescence intensity is scaled relative to the fluorescence at pH 2.0. Black and colored lines show the kinetic traces obtained by non-linear least squares fitting and by the kinetic modeling, respectively. Kinetic parameters used to reconstruct kinetic traces are shown in Table C in S1 File (elementary rate constants and kinetic *m*-values) and Table 2 (intrinsic fluorescence intensity of each structural species).

The refolding and unfolding kinetics of h-apoMb was measured by monitoring fluorescence at various urea concentrations, pH 6.0, and 8°C. The refolding reaction at pH 6.0 was initiated by mixing acid-unfolded protein (pH 2.0) with buffer containing appropriate concentrations of urea, whereas the unfolding reaction at pH 6.0 was initiated by mixing native protein (pH 6.0) with buffer containing appropriate concentrations of urea. CF and SF experiments were combined to cover the time course of folding and unfolding over the time range from ~100 μs to minutes. The dead-times of the CF and SF measurements were 102–175μs (depending on the in-house constructed mixers used in the CF experiments) and 5.3 ms, respectively. Representative kinetic traces of the refolding and unfolding reactions are shown in Fig 3. For the refolding reaction, the kinetic traces measured at urea concentrations (≤1.6 M) were fitted to a double-exponential function consisting of a faster rising phase and a slower decreasing phase whereas those at higher urea concentrations were fitted to a rising single-exponential function. The transient increase and subsequent decrease in fluorescence observed in refolding experiments at low urea concentrations reflects the rapid accumulation of a kinetic intermediate (M; see below) followed by conversion into the native state (0.1–1 s). In contrast, the increase in fluorescence without the slower decreasing phase observed at higher urea concentrations indicates that the kinetic intermediate remained populated as an equilibrium intermediate, M_eq_. The observation indicates that the kinetic intermediate was continuously converted into M_eq_ as the final urea concentration increased, and that both M and M_eq_ were formed by the same kinetic mechanism from the preceding state even under considerably different conditions (i.e., M transiently accumulated even in the absence of urea, whereas M_eq_ was stably populated even at urea concentrations higher than 2 M; see below). In addition to the kinetic phase(s) resolved by the fitting, an unresolved change in fluorescence (burst phase) was observed in the CF measurements from 0 M to 3.0 M urea, indicating accumulation of an additional intermediate, I, within the 100-μs range (see below). We also measured the refolding kinetics initiated at pH 6.0 and 0.8 M urea, where M_eq_ is a major non-native species (Fig 2D), by mixing the protein solution with buffer containing appropriate concentrations of urea in the SF device. In this case, the kinetic trace was fitted to a single-exponential function (0.13–0.53 M urea). This refolding reaction was dominated by the conversion of M_eq_ to N_eq_. The kinetic traces of the unfolding reaction were fitted to a double-exponential function at intermediate urea concentrations (3.0–4.0 M urea) and a single-exponential function at lower and higher concentrations. The transient enhancement of the fluorescence observed at 3–4 M urea (within 1 ms of unfolding) corresponds to the accumulation of a kinetic unfolding intermediate (N'; see below), which is subsequently converted into the unfolded state (0.01–0.1 s). The faster phase was not observed at low urea concentrations because of its small amplitude. It should be noted that urea was limited to ~4 M in the final conditions for CF unfolding measurement because of the solubility of urea and the 1:1 mixing ratio of the CF device.

**Fig 4:**
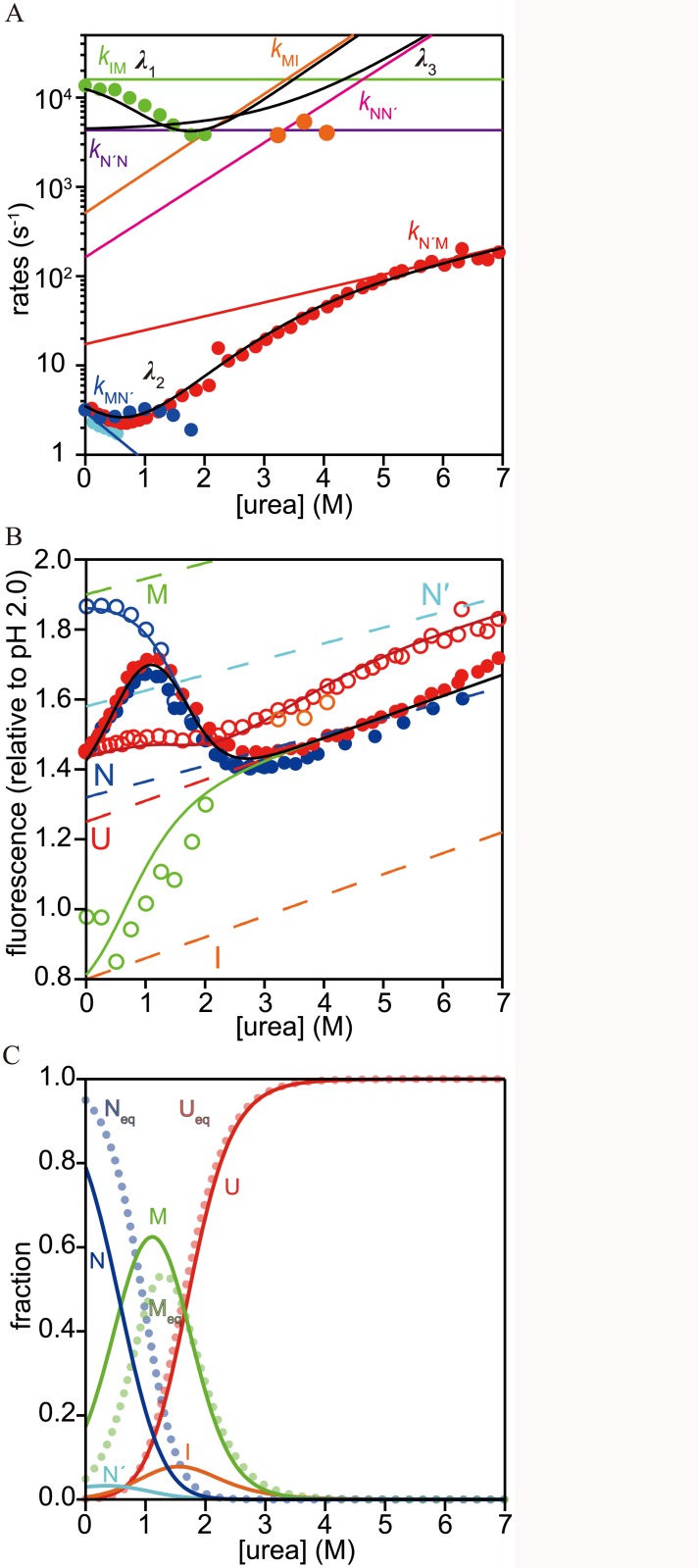
Urea-dependence of the rate constants and the cumulative amplitudes of refolding and unfolding of h-apoMb and the results obtained by the quantitative modeling. (A) Urea-dependence of the rate constants of refolding/unfolding reactions obtained in CF and SF experiments (circles): *λ*_1_ (green), *λ*_2_ (blue for refolding and red for unfolding), *λ*_2'_ (cyan), and *λ*_3_ (orange). Black and colored solid lines show the rate constants (*λ*_1_, *λ*_2_, and *λ*_3_) and elementary rate constants, respectively, predicted by the quantitative modeling (color codes for the elementary rate constants are shown). (B) The cumulative amplitudes obtained in the CF and SF experiments (circles). Open circles: *F*_0R1_ (green) and *F*_0R2_ (blue) for refolding, and *F*_0U1_ (orange) and *F*_0U2_ (red) for unfolding. Filled circles: *F*_eq_ obtained by refolding (blue) and unfolding (red). The solid lines represent *F*_eq_ (black), *F*_0R1_ (green), *F*_0R2_ (blue), *F*_0U1_ (orange), and *F*_0U2_ (red) reproduced by quantitative modeling. Here, the cumulative amplitudes are defined as *F*_0R2_ = *F*_eq_ + *F*_2_ and *F*_0R1_ = *F*_eq_ + *F*_2_ + *F*_1_ for the refolding reaction initiated at pH 2.0, and *F*_0U2_ = *F*_eq_ + *F*_2_ and *F*_0U1_ = *F*_eq_ + *F*_2_ + *F*_3_ for the unfolding reaction, where *F*_*i*_ is the amplitude of the *i*-th phase of Eq 1 and *F*_eq_ represents the fluorescence intensity at equilibrium. Dashed lines represent the fluorescence intensities of U (red), I (orange), M (green), N' (cyan), and N (blue) predicted by the quantitative modeling. (C) The fractions of each state reproduced by the quantitative modeling. The solid lines represent the reproduced fractions of U (red), I (orange), M (green), N' (cyan) and N (blue), while the dotted lines in pale colors represent the fractional U_eq_ (red), M_eq_ (green), and N_eq_ (blue) obtained by the equilibrium unfolding experiments.

Fig 4A shows the urea-dependence of the rate constants for folding and unfolding (chevron plot). For refolding initiated at pH 2.0, the slower decreasing phase (phase 2; *λ*_2_ ≈ 2–5 s^-1^) corresponds to the rate-limiting step of the overall folding reaction [48,63]. This is consistent with the finding that the rate constant of the slower unfolding phase coincides with *λ*_2_ at matching urea concentrations. The only phase observed in refolding initiated at 0.8 M urea and pH 6.0 (phase 2'; *λ*_2'_ ≈ 2–5 s^-1^) also overlaps with *λ*_2_ under matching conditions (Fig 4A), indicating that M_eq_ and the kinetic intermediate overcome a common rate-limiting step in the urea-induced folding process. On the other hand, the faster increasing phase in refolding initiated at pH 2.0 (phase 1; *λ*_1_ ≈ (0.4–1.2) × 10^4^ s^-1^) corresponds to the formation of a kinetic intermediate (M). The refolding limb exhibits a slight curvature (rollover) at low urea concentrations (~0.8 M urea), suggesting accumulation of an additional intermediate (I) within the dead-time of the CF measurements. The faster of the two unfolding phases observed at 3–4 M urea (phase 3; *λ*_3_ ≈ 4 × 10^3^ s^-1^) is distinct from *λ*_1_, indicating the formation of a kinetic unfolding intermediate (N'). The accumulation of N' also accounts for the rollover of the unfolding limb of *λ*_2_ at ~3.5 M urea. Transient accumulation of a kinetic unfolding intermediate has previously been reported during the pH-induced unfolding of sw-apoMb [52,64]. Native-like unfolding intermediates are assumed to account for the rollover of the unfolding limb for some proteins [65–67]. However, in contrast to N' (and the reported unfolding intermediate of sw-apoMb), these unfolding intermediates have negligible population during refolding and unfolding because they are always less stable than others under these conditions. The structural and kinetic properties of N' remain to be elucidated.

The observed urea-dependent amplitudes of folding and unfolding reactions provide complementary information on refolding/unfolding kinetics (Fig 4B). We considered cumulative amplitudes (*F*_0R1_, *F*_0R2_ *F*_0U1_, and *F*_0U2_) instead of individual amplitudes (*F*_*i*_ for the *i*-th phase) for the analysis of fluorescence intensity [11]. The urea-dependence of the equilibrium values, *F*_eq_, is consistent with the equilibrium unfolding transition curves already described (Fig 2). The transition curves obtained for the refolding and unfolding kinetics coincide with each other, which confirms the reversibility of the folding reactions. Because phase 2 is approximately four orders of magnitude slower than phase 1, the urea-dependence of *F*_0R2_ approximates the pre-equilibrium unfolding of M. The urea-dependence of *F*_0R2_ and *F*_eq_ shares the transition region at ~1.6 M urea, indicating that the stability of M and M_eq_ is similar. The *F*_0R1_ values are the fluorescence intensity extrapolated to *t* = 0 for CF measurements. The discrepancy between *F*_0R1_ and the fluorescence intensity expected for the unfolded state (U) is consistent with the accumulation of I as the burst phase over a urea concentration range from 0 M to ~3 M. Thus, the urea-dependence of *F*_0R1_ approximates the pre-equilibrium unfolding of I. *F*_0U2_ apparently exhibits two small but distinct transitions at ~1.6 M and ~4 M urea. The first transition arises from the unfolding of M_eq_ populated at 0 M urea (Fig 2D), whereas the second transition arises from the pre-equilibrium between the native state (N) and N'.

### Quantitative modeling of the refolding/unfolding kinetics

We quantitatively modeled the folding scheme to reproduce the observable rate constants (*λ*_1_, *λ*_2_, and *λ*_3_) and kinetic amplitudes (*F*_0R1_, *F*_0R2_, *F*_0U1_, and *F*_0U2_). As previously reported, the kinetic behavior of the folding and unfolding reactions of apoMb was consistent with a sequential mechanism rather than a parallel-pathway mechanism [5,45,47,63]. For example, a folding scheme with phases 1 and 2 located on the respective parallel pathways would not account for the enhancement of fluorescence during refolding under strongly native conditions [46]. In support of the sequential pathway mechanism, previous pulsed H/D exchange and double-jump mixing experiments using sw-apoMb revealed that the native state had not populated within a few milliseconds of the pH-induced folding reaction (see Discussion). Based on the current results for h-apoMb, at least five states were associated with its folding/unfolding. In addition to N and U, at least one intermediate (I) is responsible for the observed burst phase in the CF refolding measurements and the rollover of *λ*_1_ at ~0.8 M urea, a second intermediate (M) is responsible for phase 1, and a third intermediate (N') for phase 3 and the curvature of *λ*_2_ at ~3.5 M urea (Fig 4). Thus we assumed a sequential five-state (Scheme 2) in the quantitative modeling described below.

$$\text{U}\overset{k_{\text{UI}}}{\underset{k_{\text{IU}}}{⇌}}\text{I}\overset{k_{\text{IM}}}{\underset{k_{\text{MI}}}{⇌}}\text{M}\overset{k_{\text{MN'}}}{\underset{k_{\text{N'M}}}{⇌}}\text{N'}\overset{k_{\text{N'N}}}{\underset{k_{\text{NN'}}}{⇌}}\text{N}$$ Scheme 2

The elementary rate constants, *k*_*ij*_, are defined by Eq 2 [61]. The elementary rate constants in the absence of urea, *k* ^0^_*ij*_, and urea-dependence, *m* ^‡^_*ij*_, were manually varied to reproduce the chevron plot obtained using the rate-matrix approach. The predicted kinetic parameters are plotted in Fig 4A and listed in Table C in S1 File. As described above, phase 1 was associated with the interconversion between U, I, and M. The unfolding limb of *λ*_1_ was well approximated by *k*_MI_, whereas *k*_UI_, *k*_IU_, and *k*_IM_ were constrained by the approximate relationship *λ*_1_ ≈ *k*_UI_/(*k*_UI_ + *k*_IU_) × *k*_IM_ for the curved refolding limb. Only the ratio for *k*_UI_ and *k*_IU_ was uniquely determined because the interconversion between U and I occurred within the dead-time of the CF measurements and thus, was not directly observed. On the other hand, phases 2 and 3 were associated with the interconversion between M, N', and N. The urea-dependence of *λ*_2_ was well approximated by *k*_MN'_ and *k*_N'M_ under native conditions (the refolding limb) and strongly denaturing conditions (the unfolding limb at high urea concentrations), respectively. The elementary rate constants, *k*_NN'_ and *k*_N'N_ were determined to reproduce the unfolding limb of *λ*_2_ by the approximate relationship *λ*_2_ ≈ *k*_NN'_/(*k*_NN'_ + *k*_N'N_) × *k*_N'M_. The urea-dependence of *λ*_3_ at 3–4 M urea gave further constraint to *k*_NN'_. In addition, the fluorescence intensity of each species was optimized to reproduce the amplitudes. The fluorescence intensities of U and N were determined according to the corresponding baselines obtained experimentally. *F*_0R1_ was the fluorescence intensity of the burst phase and *F*_0R2_ approximated the fluorescence intensity transiently saturated after phase 1, in which M was most populated during refolding. Thus, under stabilizing conditions, *F*_0R1_ and *F*_0R2_ were used to determine the fluorescence intensity of I and M, respectively. *F*_0U2_ was reproduced by adjusting the fluorescence intensity of N'. The fluorescence intensity of each species is plotted in Fig 4B and listed in Table 2.

**Table 2:** Fluorescence intensity of each species (relative to that at pH 2.0) at pH 6.0 and 8°C.

*i*	Equilibrium^a^	Kinetics^b^	Slope (M^-1^)^b^
**U**	1.13	1.25	0.060
**I**	-	0.80	0.060
**M**	1.83	1.90	0.045
**N'**	-	1.58	0.045
**N**	1.24	1.32	0.045

^a^ Values were obtained by fitting a collection of the equilibrium unfolding transition curves with a global fitting algorithm to a three-state model (Scheme 1).

^b^ Values were obtained by modeling the folding/unfolding kinetics of h-apoMb based on Scheme 2.

To test the validity of the model, the obtained kinetic parameters and fluorescence intensities were used to reproduce the kinetic traces of folding and unfolding reactions at representative urea concentrations (Fig 3). The kinetic traces were in good agreement with those obtained experimentally. With 1.5 M and 2.0 M urea, small deviations were observed in the submillisecond range for the kinetic traces of refolding, which could be attributed to the fluctuation in *F*_0R1_ values (Fig 4B). Rapid decreases in fluorescence occurring within ~10 μs of refolding and ~50 μs of unfolding arose from the formation of I and the unfolding of M_eq_ populated at 0 M urea, respectively. Additionally, thermodynamic parameters calculated using kinetic parameters were in good agreement with those obtained by the fitting of equilibrium unfolding data (Table 2 and Fig 4C). Based on the fluorescence properties of each species, and because some of the species in Scheme 2 were not resolved in the equilibrium unfolding, we assigned the sum of U and I to U_eq_, the sum of N' and N to N_eq_, and M to M_eq_. Taken together, the results of the quantitative modeling accounted for the refolding/unfolding kinetics and equilibrium unfolding data obtained experimentally. Although the five-state model (Scheme 2) was consistent with the data obtained by the kinetic and equilibrium experiments, we further tested whether simpler models could account for the data. For this purpose, we explored the kinetic parameters and fluorescence intensities of each species to reproduce the folding and unfolding kinetics and unfolding equilibrium obtained by the experiments; this was achieved by assuming four-state kinetic schemes lacking one of the intermediates (I, M or N'). The urea-dependence of the rate constants, cumulative amplitudes and population of each species at equilibrium obtained via the quantitative modeling assuming the four-state models are shown in Fig D in S1 File. The discrepancy between the results obtained in the experiments and in the quantitative modeling (assuming a four-state model) lends support to the validity of the five-state kinetic scheme (Scheme 2).

## Discussion

### Relationship between the kinetic intermediate M and the equilibrium intermediate M_eq_

The relationship between kinetic and equilibrium intermediates has been investigated for several proteins by comparing the structural and thermodynamic properties of the two types of states [3–11]. Such comparative studies have been extensively conducted, in most cases to assess the intermediates of apoMb for pH-induced folding and unfolding. H/D exchange NMR studies on sw-apoMb showed that the equilibrium state(s) populated at pH 4 and a transient kinetic intermediate formed in ~10 ms of refolding at pH 6 exhibited similar patterns of protection, i.e., both intermediates contained native-like secondary structures in the A-, G- H-, and part of the B-, helices [5–7,29,34]. For h-apoMb, time-resolved CD and small angle X-ray scattering (SAXS) experiments revealed that the helix content (~40% compared with the native state) and overall molecular size (~30% larger radius of gyration, *R*_g_, than the native state) were also similar for a kinetic intermediate (I_2_) and a trichloroacetate stabilized equilibrium intermediate [48]. Additionally, a slow folding phase with similar rate and amplitude was observed in folding experiments on sw-apoMb starting from either fully unfolded state or the partially folded state at pH 4, indicating that these intermediates were converted into the native state via a common kinetic mechanism [46]. For urea-induced folding of apoMb, Samatova *et al*. measured urea-dependence of the folding/unfolding of sw-apoMb and the variants using stopped-flow fluorescence under conditions similar to those used in the present study (pH 6.2 and 11°C) [44]. They measured the apparent rate constants of the rate-limiting step of refolding over a wide range of urea concentrations, which was most likely to correspond to *λ*_2_ in the present study (Fig 4A), and the burst phase of refolding (the fluorescence change that occurred within the dead time of the stopped-flow apparatus (<< 20 ms)). The burst phase in their study was likely to correspond to *F*_0R2_ (Fig 4B) in our study. However, the formation of kinetic intermediates and non-native equilibrium states has not been directly compared under a wide range of conditions from strongly native to denaturing, in part due to the limited time resolution of conventional stopped-flow methods.

**Fig 5:**
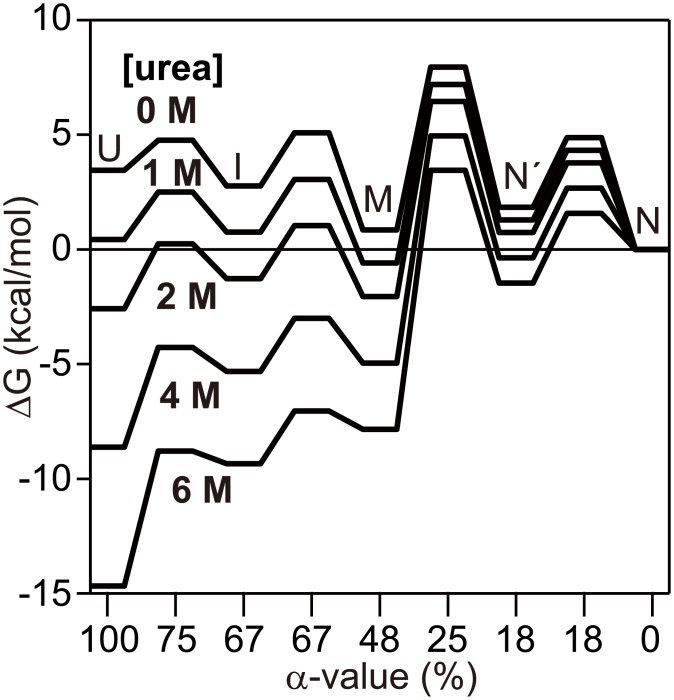
A free energy diagram of h-apoMb at pH 6.0 and 8°C as a function of α-value (i.e., the change in the solvent accessible surface area relative to N).

In this study, we showed that the same set of kinetic barriers were encountered in the formation of the kinetic intermediate (M) and the corresponding equilibrium state (M_eq_) for urea-induced folding and unfolding. In particular, we showed, using CF and SF methods (Fig 4), that M shifts from a transient intermediate to a well populated equilibrium state as the urea concentration is increased. In the refolding reaction, M transiently accumulated during refolding at pH 6.0 and 0 M urea, and formation of M was continuously observed in the refolding up to 2.2 M urea as long as the fast phase *λ*_1_ had measurable amplitude. In contrast, the formation of N was observed only at <~1.6 M urea due to the low stability of N (the free energy difference between N and M is ~1.0 kcal/mol at pH 6.0 and 0 M urea; see Table 1). It follows that M predominantly accumulates as an equilibrium intermediate between 0.7 and 2.0 M urea, a urea concentration range in which N is no longer populated predominantly at equilibrium. More intuitively, at low urea concentrations, M was preferentially formed over I because *k*_IM_ >> *k*_MI_, and N', which is readily converted into N, was preferentially formed over M because *k*_MN'_ is larger than the apparent unfolding rate constant (*λ*_2_ ≈ *k*_NN'_/(*k*_NN'_ + *k*_N'N_) × *k*_N'M_); this leads to the conversion of U to N with transient accumulation of M as a kinetic intermediate. In contrast, at 0.7–2 M urea, M was preferentially formed not only over N' because *λ*_2_ was larger than *k*_MN'_ but also over I because *k*_IM_ >> *k*_MI_; thus, M is stably populated as an equilibrium state, which is equivalent to M_eq_. Therefore, M and M_eq_ are formed by overcoming the same set of kinetic barriers, consistent with a single molecular species. Furthermore, the stability of N relative to M determines whether M transiently accumulates during refolding under strongly native conditions (~0 M urea) or is populated at equilibrium under moderately denaturing conditions (0.7–2.0 M urea). Moreover, the matching urea dependence of *λ*_2_ and *λ*_2_' indicates that M and M_eq_ is converted into N via the same kinetics process, as previously reported for the pH-induced folding of sw-apoMb [46]. Quantitative modeling also revealed that M and M_eq_ had similar thermodynamic stabilities and fluorescence intensities (Fig 3A, Tables 1 and 2). This is supported by the shared transition region of *F*_0R2_ and *F*_eq_ at ~1.6 M urea because the urea dependence of *F*_0R2_ represented the pre-equilibrium unfolding of M. The energetics of the (un)folding reaction are schematically illustrated by a free energy diagram as a function of α-value, a measure of the change in solvent-accessible surface area occurring during folding. Fig 5 shows that M is one of the most stable species at 1–2 M urea, which leads to the accumulation of M as an equilibrium intermediate. This implies that regions that are more robust to perturbation by urea form at an earlier stage of folding, which further supports the validity of the use of equilibrium intermediates as counterparts for kinetic intermediates. It should be noted that formation of the overall native-like structure (i.e., folding of the C-, D-, E- and part of the B-helix regions that are less stable than the A-, G-, and H-helix regions) would occur during the M → N conversion (phase 2) considering the similarity in the rate-limiting step of folding between h-apoMb and sw-apoMb.

### Burst phase intermediate, I

A kinetic intermediate, I, accumulates during folding within the dead-time of CF measurements, as indicated by the rollover in *λ*_1_ and the missing amplitude in refolding experiments at low urea concentrations. Quantitative modeling showed that the I state has lower fluorescence than U (0.80 vs. 1.25) and an α-value closer to that of M than U (Fig 5). These results suggest the overall structure of I is already rather compact, consistent with the observation that a considerable decrease in *R*_g_ (~6 Å) occurs within ~300 μs of refolding [48].

Our results are also supported by a recent study on the folding of the apo-form of the heme-binding domain of flavohemoglobin (apoHmpH) from *Escherichia coli*, a protein with a globin fold structurally analogous to h-apoMb [68]. ApoHmpH, which has a single-Trp at position 120 in the H-helix, forms a kinetic intermediate during folding on the 10-μs time scale. Because the fluorescence spectrum of the intermediate is blue-shifted compared to that of the unfolded state, the observed fluorescence quenching is attributed to the establishment of specific short-range intramolecular interactions with quenching residues located in the core region around the Trp residue. This quenched and blue-shifted fluorescence of apoHmpH I-state is consistent with the reduced fluorescence intensity of I observed in our study. Although they are located in different helices, the Trp residues of both proteins are involved in the core region consisting of A-, G-, and H-helices. Thus, the change in Trp fluorescence that occurs during early folding is likely to represent similar structural events for both proteins. In addition, infrared (IR) spectroscopy and UV resonance Raman (UVRR) scattering studies on h-apoMb indicate that both solvated and buried helices are present, but the side-chains of Trp residues are partially solvent-shielded over a time scale of 100 μs [63,69]. Thus, both of these globins rapidly form an intermediate with a quenched and blue-shifted fluorescence spectrum consistent with a collapsed state with a partially formed A-, (B-), G-, and H-helix core.

### Folding mechanism of h-apoMb

We showed that a sequential five-state mechanism (Scheme 2) quantitatively accounts for the three observable kinetic phases along with the burst phase in refolding (Figs 3 and 4). Multiphasic folding/unfolding kinetics is, however, also observed for a parallel-pathway folding mechanism, as mentioned above. Here, we discuss the folding mechanism that appropriately represents the folding of h-apoMb. Previous pulsed H/D experiments combined with mass spectrometry on sw-apoMb folding [45, 70] showed that the pH-induced kinetic folding intermediate had already accumulated within 6 ms of initiating folding without detectable amounts of the native state, whereas the native state appeared only after 100 ms of folding at the expense of the intermediate state. This indicates that the folding of sw-apoMb is consistent with a sequential rather than a parallel-pathway mechanism. The sequential mechanism is also supported as a folding mechanism for h-apoMb as indicated by the results of previous continuous-flow CD and SAXS experiments [48]. In these studies, two kinetic phases with time constants of 5 and 49 ms were detected along with a burst phase (dead-time of 280 μs) during pH-induced refolding from pH 2.2 to pH 6.0. The observation that *R*_g_ remained constant (23.7 Å) from 300 μs to ~10 ms suggested that h-apoMb folds to the native state via a sequential mechanism with accumulation of two folding intermediates; in contrast, a parallel-pathway mechanism would predict a considerable reduction of *R*_g_ over this time range, since both pathways lead to the compact native state. The observed stepwise increase in α-helical content and reduction in *R*_g_ in each kinetic phase further support sequential accumulation of two folding intermediates. In addition, a previous ultra-fast H/D exchange experiment on sw-apoMb [5] revealed that the amide protons in the A-, G-, and H-helix regions of the native structure were already protected within 400 μs of pH-induced folding followed by protection of amide protons in the B-, C-, and E-helix around 6 ms. Taken together, the folding of h-apoMb is most likely represented by a sequential rather than a parallel-pathway mechanism, assuming that the folding mechanisms are conserved between sw-apoMb and h-apoMb.

A stepwise change in structure was also observed in previous molecular dynamics simulations [49–51]. Unfolding simulations induced by protonation of ionizable groups, which corresponds to acid unfolding, indicated that sw-apoMb unfolds via partially unfolded structures similar to the pH 4 intermediate, i.e., before it reaches a fully unfolded state, A-, G-, H- and part of B-helices form a compact core with helix contents consistent with those obtained in an experimental study [33]. Thermal unfolding simulations of myoglobins, including sperm whale and horse myoglobins, also demonstrated transient formation of partially unfolded structures with a few helix regions partially formed during thermal unfolding at 500 K (although helix regions relevant to heme binding unfolded more slowly than the apo-proteins due to the presence of heme). In these simulation studies, partially unfolded structures were observed, which suggests that this protein folds via a sequential mechanism.

In Scheme 2, the three intermediates were assumed as on-pathway states, i.e., they were productive intermediates. Here, we also consider the possibility that an intermediate is a dead-end product under the assumption of a five-state sequential scheme. In this case, the intermediate is not productive and should be at least partially unfolded to reach the native state. We therefore ask whether I or M could be an off-pathway intermediate as follows:

I (M)⇌U⇌M (I) ⇌N'⇌N Scheme 3

The kinetic behavior predicted by Scheme 3 is essentially indistinguishable from that of Scheme 2. This is because pre-equilibrium among U, I, and M is established very rapidly (within ~ms), whereas the conversion to N' becomes significant where the time scale is 100-ms to seconds. The difference between these schemes could be kinetically distinguished if the U ↔ I interconversion was not only experimentally observed but also strongly coupled with the I ↔ M interconversion, as previously reported for the folding of immunity protein 7 [71]. However, the α-values of I and M are expected to be ~50% according to the above schemes, which indicates that I/M should be globally unfolded before the conversion into N within the submillisecond range. This is unlikely when considering that the native-like substructure is already formed on the 400-μs time scale of refolding of the homologous sw-apoMb [5]. Thus, we propose that Scheme 2 represents the folding of h-apoMb but acknowledge that further improvements in time resolution will likely provide additional insight into the folding of this protein.

We observed the formation of a single folding intermediate (M) in the submillisecond-to-millisecond range during refolding. In contrast, some previous kinetic studies of both h-apoMb and sw-apoMb reported two folding intermediates that resulted in sequential four-state kinetic schemes [46,48]. According to previous CD, SAXS, and IR absorption-detected CF experiments on h-apoMb [48,63], the two intermediates exhibited similar secondary structure and overall size. Additionally, the intermediates of sw-apoMb (Ia/Ib) also had similar fluorescence intensities, although UVRR-detected CF measurements of h-apoMb suggested differences in the local environment of the two Trp side-chains [69]. Thus, even if the kinetic intermediate ensemble (M) observed for h-apoMb in this study consisted of multiple forms (such as Ia and Ib), their fluorescence intensities might be similar in the case of h-apoMb, which would make it difficult to resolve them via the Trp fluorescence measurements employed here. Another possibility is that I and M corresponded to Ia and Ib, respectively. As already discussed, it is likely that M involves Ib because the folding from these intermediates is the rate-limiting step and the rate constants (*λ*_2_ ≈ 2 s^-1^ at 0 M urea, pH 6.0, and 8°C) are comparable to those obtained in previous studies (~5 s^-1^ for sw-apoMb at pH 6.0 and 5°C; ~20 s^-1^ for h-apoMb at pH 6.0 and 26°C) given the differences in sequence and temperature. However, the rate constant of the conversion from I to M (*λ*_1_ ≈ 10^4^ s^-1^ at 0 M urea, pH 6.0, and 8°C) was two-to-three orders of magnitude larger than the rate constants of the conversion between Ia and Ib (~ 50 s^-1^ for sw-apoMb at pH 6.0 and 5°C; ~200 s^-1^ for h-apoMb at pH 6.0 and 26°C). This difference is too large to confidently assign phase 1 to the conversion from Ia to Ib. The I ↔ M interconversion is more likely to correspond to conversion of the burst phase intermediate to a folding intermediate observed in previous studies of pH-induced folding of h-apoMb using IR absorption- and UVRR-detected CF [63, 69]. In fact, the burst phase intermediates and I were formed within a similar time range (<100–200 μs), and the folding intermediate formed was indicated to correspond to Ia when observed by IR. However, their rate constants were 2.5−3×10^3^ s^-1^ at 20–25°C, which are much slower than those obtained in this study considering the temperature used here (8°C). Nevertheless, the I ↔ M interconversion is more likely to correspond to the kinetics detected in the submillisecond time range by the IR and UVRR measurements than the Ia ↔ Ib interconversion, although detailed investigation is needed to understand the early folding events of apoMb.

This study is complementary to a recent study on the folding/unfolding kinetics of the intermediates of sw-apoMb at pH 4, since the pH 4 intermediate is analogous to M_eq_, based on its spectroscopic properties (Tables A and B, and Fig B in S1 File) [52]. Two intermediates were observed in urea-induced folding/unfolding reactions at pH 4.2, including one that accumulates with a time constant of ~30 μs during refolding at low urea concentrations, and a second one formed within ~200 μs of unfolding at high urea concentrations. Assuming that the pH 4 intermediate of sw-apoMb corresponds to the M_eq_ of this study, the counterpart of the burst phase intermediate, I, would be the sw-apoMb refolding intermediate. Indeed, even at pH 4.2 the sw-apoMb refolding intermediate accumulated in the 100-μs range (comparable to the dead-time of the CF device used in this study), which would be observed as the burst phase intermediate during refolding under native conditions at pH 6.0. On the other hand, there is a distinct difference in the fluorescence properties between these two intermediates. The I state of h-apoMb is less fluorescent than U, whereas the sw-apoMb folding intermediate exhibits a higher fluorescence intensity compared to U. Nevertheless, because fluorescence properties often reflect the local as well as global environment around Trp residues, the sw-apoMb refolding intermediate is still likely to correspond to I based on the similarity in their kinetic behavior. In addition, it should be noted that preliminary CF unfolding measurements initiated at 0.8 M urea and pH 6.0, where M is populated, detected changes in fluorescence within the dead-time of the device, and that unfolding was much faster than the reverse I → M conversion (~10^4^ s^-1^). The non-two-state behavior implies transient accumulation of an unfolding intermediate, which is consistent with the observation of the unfolding intermediate of sw-apoMb, although further investigation is required.

In summary, we systematically investigated the urea-dependence of the refolding/unfolding kinetics and unfolding equilibrium of h-apoMb, and found that five species (U, I, M, N', and N) are associated with the refolding/unfolding reactions. We quantitatively modeled the data using a five-state sequential kinetic model. The formation of M is observed over a wider range of urea concentrations (0–2.2 M) than the formation of N (0–1.6 M). Based on the differences between the urea-dependent stability of M and N, we determined that M and M_eq_ are formed by overcoming a shared kinetic barrier in the folding reaction and that the stability of N relative to M determines whether M transiently accumulates during folding or is populated as M_eq_. An additional intermediate, I, accumulated within the dead-time of refolding, and the native-like unfolding intermediate, N', accumulated in <1 ms of unfolding.

## Supporting Information

S1 File Supporting information related to “Evidence for a Shared Mechanism in the Formation of Urea-Induced Kinetic and Equilibrium Intermediates of Horse Apomyoglobin from Ultrarapid Mixing Experiments.” S1 File contains the following sections. Methods: description of the methods used for detailed analysis of the quantitative modeling; Table A: helix contents of N_eq_, M_eq_, and U_eq_ estimated by ellipticity at 222 nm and of a crystal structure; Table B: differences in the primary structures and helix propensity values between h-apoMb and sw-apoMb; Table C: kinetic parameters estimated by monitoring the refolding and unfolding kinetics of h-apoMb based on Scheme 2 at pH 6.0 and 8°C; Fig A: temperature dependence of the ellipticity at 222 nm in 12 mM sodium citrate at pH 6.0; Fig B: far-UV CD and fluorescence spectra of h-apoMb in 12 mM sodium citrate at 8°C under various conditions; Fig C: time-dependent changes in fluorescence during the folding of h-apoMb at pH 6.0 and 0.8 M urea compared with those initiated at pH 2.0; Fig D: urea-dependence of the rate constants and the cumulative amplitudes of refolding and unfolding, and population of each species of h-apoMb calculated by the quantitative modeling assuming four-state schemes.
(PDF)
