# Supplementary material for: Evidence for a Shared Mechanism in the Formation of Urea-Induced Kinetic and Equilibrium Intermediates of Horse Apomyoglobin from Ultrarapid Mixing Experiments
Source: PLoS One. 2015 Aug 5;10(8):e0134238. doi: 10.1371/journal.pone.0134238 (PMC4526358; doi:10.1371/journal.pone.0134238)
Supplement: S1 File — contains the following sections. Methods: description of the methods used for detailed analysis of the quantitative modeling; Table A: helix contents of Neq, Meq, and Ueq estimated by ellipticity at 222 nm and of a crystal structure; Table B: differences in the primary structures and helix propensity values between h-apoMb and sw-apoMb; Table C: kinetic parameters estimated by monitoring the refolding and unfolding kinetics of h-apoMb based on Scheme 2 at pH 6.0 and 8°C; Fig A: temperature dependence of the ellipticity at 222 nm in 12 mM sodium citrate at pH 6.0; Fig B: far-UV CD and fluorescence spectra of h-apoMb in 12 mM sodium citrate at 8°C under various conditions; Fig C: time-dependent changes in fluorescence during the folding of h-apoMb at pH 6.0 and 0.8 M urea compared with those initiated at pH 2.0; Fig D: urea-dependence of the rate constants and the cumulative amplitudes of refolding and unfolding, and population of each species of h-apoMb calculated by the quantitative modeling assuming four-state schemes. (PDF) [file pone.0134238.s001.pdf]

Supporting Information: S1 File

Evidence for a Shared Mechanism in the Formation of Urea-Induced Kinetic and Equilibrium Intermediates of Horse Apomyoglobin from Ultrarapid Mixing Experiments

Takuya Mizukami[1,#], Yukiko Abe[1], Kosuke Maki[1*]

[1] Graduate School of Science, Nagoya University, Nagoya, Aichi 464-8602, Japan

[#] Current Address: Fox Chase Cancer Center, Philadelphia, PA 19111, USA

Methods: Details of quantitative analysis

The time evolution of reactants and products represented by a reaction scheme are obtained by solving a series of rate equations in differential forms (given the scheme, the associated elementary rate constants, and the initial condition). For the five-state sequential folding of h-apoMb, the scheme is represented as follows:

$$\mathrm{U} \underset{k_{\mathrm{IU}}}{\overset{k_{\mathrm{UI}}}{\rightleftharpoons}} \mathrm{I} \underset{k_{\mathrm{MI}}}{\overset{k_{\mathrm{IM}}}{\rightleftharpoons}} \mathrm{M} \underset{k_{\mathrm{N'M}}}{\overset{k_{\mathrm{MN'}}}{\rightleftharpoons}} \mathrm{N}' \underset{k_{\mathrm{NN'}}}{\overset{k_{\mathrm{N'N}}}{\rightleftharpoons}} \mathrm{N}. \qquad \text{Scheme 2}$$

Eq. S1 represents the rate equations:

$$\frac{\mathrm{d}}{\mathrm{d}t}\begin{bmatrix}[\mathrm{U}](t)\\ [\mathrm{I}](t)\\ [\mathrm{M}](t)\\ [\mathrm{N'}](t)\\ [\mathrm{N}](t)\end{bmatrix} = \begin{bmatrix} -k_{\mathrm{UI}} & k_{\mathrm{IU}} & 0 & 0 & 0\\ k_{\mathrm{UI}} & -k_{\mathrm{IU}}-k_{\mathrm{IM}} & k_{\mathrm{MI}} & 0 & 0\\ 0 & k_{\mathrm{IM}} & -k_{\mathrm{MI}}-k_{\mathrm{MN'}} & k_{\mathrm{N'M}} & 0\\ 0 & 0 & k_{\mathrm{MN'}} & -k_{\mathrm{N'M}}-k_{\mathrm{N'N}} & 0\\ 0 & 0 & 0 & k_{\mathrm{N'N}} & -k_{NN'}\end{bmatrix}\begin{bmatrix}[\mathrm{U}](t)\\ [\mathrm{I}](t)\\ [\mathrm{M}](t)\\ [\mathrm{N'}](t)\\ [\mathrm{N}](t)\end{bmatrix}, \qquad \text{Eq. S1}$$

where [X] is the concentration of species X (X = U, I, M, N', and N) at time t, and k_{ij} is the elementary rate constant of the i $\rightarrow$ j conversion (i, j = U, I, M, N', and N). The solution to Eq. S1 is represented as follows:

$$\mathbf{X}(t) = \exp(\mathbf{K}t)\mathbf{X}(0), \qquad \text{Eq. S2}$$

where

$$\mathbf{X}(t) = \begin{bmatrix}[\mathrm{U}](t)\\ [\mathrm{I}](t)\\ [\mathrm{M}](t)\\ [\mathrm{N'}](t)\\ [\mathrm{N}](t)\end{bmatrix}, \mathbf{K} = \begin{bmatrix} -k_{\mathrm{UI}} & k_{\mathrm{IU}} & 0 & 0 & 0\\ k_{\mathrm{UI}} & -k_{\mathrm{IU}}-k_{\mathrm{IM}} & k_{\mathrm{MI}} & 0 & 0\\ 0 & k_{\mathrm{IM}} & -k_{\mathrm{MI}}-k_{\mathrm{MN'}} & k_{\mathrm{N'M}} & 0\\ 0 & 0 & k_{\mathrm{MN'}} & -k_{\mathrm{N'M}}-k_{\mathrm{N'N}} & 0\\ 0 & 0 & 0 & k_{\mathrm{N'N}} & -k_{\mathrm{NN'}}\end{bmatrix} \qquad \text{Eq. S3}$$

and

$$\exp(\mathbf{K}t) = \sum_{n=0}^{\infty}\frac{1}{n!}(\mathbf{K}t)^n = \mathbf{E} + \mathbf{K}t + \frac{(\mathbf{K}t)^2}{2!} + \cdots. \qquad \text{Eq. S4}$$

The rate matrix **K** is diagonalized by a matrix **P** consisting of a set of eigenvectors as follows:

$$\mathbf{P}^{-1}\mathbf{K}\mathbf{P} = \begin{bmatrix} -\lambda_1 & 0 & 0 & 0 & 0 \\ 0 & -\lambda_2 & 0 & 0 & 0 \\ 0 & 0 & -\lambda_3 & 0 & 0 \\ 0 & 0 & 0 & -\lambda_4 & 0 \\ 0 & 0 & 0 & 0 & 0 \end{bmatrix} \equiv -\mathbf{\Lambda}, \qquad \text{Eq. S5}$$

where λ_1, λ_2, λ_3, and λ_4 are non-zero eigenvalues of the rate matrix **K** and the fifth eigenvalue is zero. By using the matrices **P** and –**Λ**, the time-evolution of the concentration of each species (Eq. S2) is represented as follows:

$$\mathbf{X}(t) \equiv \begin{bmatrix} [\mathrm{U}](t) \\ [\mathrm{I}](t) \\ [\mathrm{M}](t) \\ [\mathrm{N'}](t) \\ [\mathrm{N}](t) \end{bmatrix} = \mathbf{P}\exp(-\mathbf{\Lambda}t)\mathbf{P}^{-1}\mathbf{X}(0) = \mathbf{P}\begin{bmatrix} e^{-\lambda_1 t} & 0 & 0 & 0 & 0 \\ 0 & e^{-\lambda_2 t} & 0 & 0 & 0 \\ 0 & 0 & e^{-\lambda_3 t} & 0 & 0 \\ 0 & 0 & 0 & e^{-\lambda_4 t} & 0 \\ 0 & 0 & 0 & 0 & 0 \end{bmatrix}\mathbf{P}^{-1}\mathbf{X}(0). \qquad \text{Eq. S6}$$

Because the concentration of each species varies as a function of the sum of four exponentials plus a constant according to Eq. S6, the non-zero eigenvalues correspond to the apparent rate constants.

In this study, the apparent rate constants ($\lambda_{1\text{-}4}$) were searched by manually varying the elementary rate constants as inputs to fully account for their urea-dependence (see the chevron plot in Figure 4A) and fractions of each species at equilibrium (see Figure 4C). Here, λ_4 was assigned to the apparent rate of the U $\rightleftarrows$ I interconversion, which was too fast to resolve as a kinetic phase and was therefore only detected as the burst phase in the experiments. The urea concentration dependence of the elementary constants was assumed to follow Eq. S7 (Eq. (2) in the main text):

$$\ln k_{ij}([\text{urea}]) = \ln k_{ij}^{0} + \frac{m_{ij}^{\ddagger}[\text{urea}]}{RT}, \qquad \text{Eq. S7}$$

where k_{ij}([urea]) and k_{ij}^{0} are the elementary constants for the $i \rightarrow j$ transition at a urea concentration ([urea]) and 0 M urea, respectively, and $m^{\ddagger}_{ij}$ is the kinetic *m*-value that corresponds to the slope. *R* and *T* are the gas constant and absolute temperature, respectively. In addition, the fluorescence-monitored kinetic trace ($F_{obs}(t)$) was calculated as follows (see Figure 3):

$$F_{obs}(t) = F_{\mathrm{U}} \times \frac{[\mathrm{U}](t)}{C_0} + F_{\mathrm{I}} \times \frac{[\mathrm{I}](t)}{C_0} + F_{\mathrm{M}} \times \frac{[\mathrm{M}](t)}{C_0} + F_{\mathrm{N'}} \times \frac{[\mathrm{N'}](t)}{C_0} + F_{\mathrm{N}} \times \frac{[\mathrm{N}](t)}{C_0}, \qquad \text{Eq. S8}$$

where F_{U}, F_{I}, F_{M}, $F_{\mathrm{N'}}$, and F_{N} are the fluorescence intensity of U, I, M, N', and N, respectively, and C_0 is the total protein concentration. Eq. S8 allowed us to calculate the (cumulative) amplitudes of each phase as a function of urea concentration (see Figure 4B). The kinetic parameters and the fluorescence intensities used in this study are listed in Table C in S1 File (below) and Table 2 (in the main text), respectively.

Although the parameters listed in Table C and Table 2 can be obtained in principle by using the aforementioned method, we describe (below) the key features of the urea-induced folding/unfolding of h-apoMb to give constraints to the kinetic parameters in the practical analysis. These features are as follows: 1) the sigmoidal feature of the burst phase, 2) the rollover of λ_1 at ~1 M urea, and 3) the detection of λ_3 and rollover of λ_2 at 3–4 M urea; these allow us to find appropriate values of k_{UI}, k_{IU}, k_{IM}, $k_{\mathrm{N'M}}$, $k_{\mathrm{N'N}}$, and $k_{\mathrm{NN'}}$. The other two elementary rate constants, k_{MI} and $k_{\mathrm{MN'}}$, were determined from the unfolding limb of λ_1 and the refolding limb of λ_2, respectively. The fluorescence intensity of each species was determined so that the cumulative amplitudes were reproduced.

(1) The sigmoidal feature of the burst phase ($F_{0\mathrm{R1}}$). At low urea concentrations, $F_{0\mathrm{R1}}$ depends on urea concentration as follows:

$$F_{0R1}([\mathrm{urea}]) \approx \frac{k_{\mathrm{IU}}}{k_{\mathrm{UI}} + k_{\mathrm{IU}}} F_{\mathrm{U}} + \frac{k_{\mathrm{UI}}}{k_{\mathrm{UI}} + k_{\mathrm{IU}}} F_{\mathrm{I}}, \qquad \text{Eq. S9}$$

Because pre-equilibrium is attained between U and I within the dead time of the CF measurements, Eq. S9 approximates a two-state unfolding transition curve and accounts for the sigmoidal feature of $F_{0\mathrm{R1}}$. The ratio of k_{UI} and k_{IU} was adjusted to reproduce $F_{0\mathrm{R1}}$ with the midpoint of the transition at ~1 M urea.

(2) The rollover of λ_1 at ~1 M urea. At low urea concentrations, λ_1 was approximated as a function

of urea concentration by

$$\lambda_1([\text{urea}]) \approx \frac{k_{\text{UI}}}{k_{\text{UI}} + k_{\text{IU}}} k_{\text{IM}} \,. \qquad \text{Eq. S10}$$

Eq. S10 clearly shows that λ_1 depends on the equilibrium constant of the U $\rightleftarrows$ I transition, $K_{\text{UI}} \equiv k_{\text{IU}}/k_{\text{UI}}$, and decreases more steeply than the linearly decreasing k_{IM}, which reflects the destabilization of I as urea concentration increases. The rollover is significant around the midpoint of the transition at ~1 M urea. k_{IM} was adjusted to reproduce the rollover based on the appropriate k_{UI} and k_{IU} values.

(3) Detection of λ_3 and rollover of λ_2 at 3–4 M urea. Phase 3 (λ_3) is experimentally detected within a limited range of 3–4 M urea because λ_3 is too fast to resolve with the CF measurements above 4 M urea and the unfolding intermediate N', which is responsible for λ_3, is too unstable to even transiently accumulate below 3 M urea. It follows that the elementary rate constants relevant to λ_3, $k_{\text{NN'}}$, and $k_{\text{N'N}}$ are similar at ~3 M urea; thus, $k_{\text{NN'}} > k_{\text{N'N}}$ and $k_{\text{NN'}} < k_{\text{N'N}}$ above and below ~3 M urea, respectively. The urea concentration dependence of the elementary rate constants accounts for the rollover of λ_2 found at ~3 M urea because λ_2 is approximated above ~1 M urea as follows:

$$\lambda_2([\text{urea}]) \approx \frac{k_{\text{NN'}}}{k_{\text{NN'}} + k_{\text{N'N}}} k_{\text{N'M}} \,, \qquad \text{Eq. S11}$$

Thus, $k_{\text{N'N}}$ and $k_{\text{NN'}}$ were adjusted to reproduce λ_3, whereas $k_{\text{N'M}}$ was adjusted to reproduce λ_2, according to Eq. S11.

Table A: Helix contents of N_{eq}, M_{eq} and U_{eq} estimated from ellipticity values at 222nm and of the crystal structure

State	Holo	N_{eq}		M_{eq}			U_{eq}		
condition	Crystal (1AZI)	pH 6.0	Global Fitting	pH 4.0	1.2 M urea	Global Fitting	pH2.0	8 M urea	Global Fitting
$[\theta]_{222}$ (deg cm^2 dmol^{-1})		-19,932 [a]	-20,750 [b]	-13,984 [a]	-11,152 [a]	-12,364 [b]	-4,349 [a]	+385 [a]	-3,896 [b]
f_h^c		0.58	0.61	0.38	0.29	0.33	0.07	-0.09	0.05
f_h^d		0.62	0.64	0.43	0.34	0.38	0.13	-0.02	0.12
f_h^e		0.59	0.61	0.42	0.35	0.38	0.14	0.02	0.13
f_h^f		0.63	0.65	0.45	0.37	0.40	0.15	0.02	0.14
f_h^g	0.78								

[a] Ellipticity values were measured at 8°C (Figure 2A (main text) and Figure S2A (File S1)).

[b] Ellipticity values were estimated by the global fitting of urea-induced unfolding transition curves measured at pH 6.0 and 8°C (Figure 2A (main text) and Figure S2A (File S1)).

[c] Helical contents were estimated using an equation by Chen YH *et al.* [1] without temperature correction, where $[\theta]_{222}$ for residues in helix is -32,640 deg cm^2/dmol and $[\theta]_{222}$ for residues in coil is -2,340 deg cm^2/dmol. The edge effects are not considered.

[d] Helical contents were estimated using an equation by Sabelko J *et al.* [2] without temperature correction, where $[\theta]_{222}$ for residues in helix is -32,100 deg cm^2/dmol and $[\theta]_{222}$ for residues in coil is -200 deg cm^2/dmol. The edge effects are not considered.

[e] Helical contents were estimated using an equation by Dasmeh P & Kepp KP [3] without temperature correction, where $[\theta]_{222}$ for residues in helix is -39,500 deg cm^2/dmol, $[\theta]_{222}$ for residues

in coil is +1,000 deg cm^2/dmol, $[\theta]_{222}$ for the 2.57 residues at the edge of helix is zero and helix numbers found in N, M, and U states are 5, 4, and 2, respectively.

[f] Helical contents were estimated using the equation by Dasmeh P & Kepp KP[3] without temperature correction, where $[\theta]_{222}$ for residues in helix is -36,800 deg cm^2/dmol instead of -39,500 deg cm^2/dmol.

[g] Helical structures were assigned by DSSP based on a crystal structure (PDB: 1AZI).

Table B: Difference in the primary structures and helix propensity values between h- and sw-apoMb.

Secondary struct.	A-helix				
Residue	1	4	9	12	15
H-apoMb	Gly	Asp	Gln	Asn	Gly
(helix propensity[a])	(0.43)	(0.99)	(1.27)	(0.76)	(0.43)
Sw-apoMb	Val	Glu	Leu	His	Ala
(helix propensity[a])	(0.98)	(1.59)	(1.34)	(1.05)	(1.41)

Secondary struct.	B-helix				
Residue	21	27	28	34	35
H-apoMb	Ile	Glu	Val	Thr	Gly
(helix propensity)	(1.09)	(1.59)	(0.98)	(0.76)	(0.43)
Sw-apoMb	Val	Asp	Ile	Lys	Ser
(helix propensity)	(0.98)	(0.99)	(1.09)	(1.23)	(0.57)

Secondary struct.	E-helix			
Residue	45	66	67	74
H-apoMb	Lys	Thr	Val	Gly
(helix propensity)	(1.23)	(0.76)	(0.98)	(0.43)
Sw-apoMb	Arg	Val	Thr	Ala
(helix propensity)	(1.21)	(0.98)	(0.76)	(1.41)

Secondary struct.	G-helix		H-helix		
Residue	109	118	132	140	151
H-apoMb	Asp	Lys	Thr	Asn	Phe
(helix propensity)	(0.99)	(1.23)	(0.76)	(0.76)	(1.16)
Sw-apoMb	Glu	Arg	Asn	Lys	Tyr
(helix propensity)	(1.59)	(1.21)	(0.76)	(1.23)	(0.74)

[a] Helix propensity values were calculated according to Williams RW *et al.* [4]

Table C: Kinetic parameters estimated by monitoring the refolding and unfolding kinetics of h-apoMb based on Scheme 2 at pH 6.0 and 8°C.

i j	k^0_{ij} (s^{-1})[a]	$m^{\ddagger}_{ij}$ (kcal/mol/M)[b]
U I[c]	9.6×10^4	-0.76
I U[c]	2.8×10^4	0.24
I M	1.6×10^4	0.00
M I	5.1×10^2	0.57
M N'	3.0	-0.70
N' M	1.7×10^1	0.20
N' N	4.3×10^3	0.00
N N'	1.6×10^2	0.55

[a] k^0_{ij} indicates the elementary rate constant for a given process in the absence of urea.

[b] Kinetic *m*-values, $m^{\ddagger}_{ij}$, were estimated according to Eq. (2).

[c] Only the ratio k_{UI}/k_{IU} is uniquely determined.

Fig A: Temperature dependence of the ellipticity at 222 nm in 12 mM sodium citrate at pH 6.0.

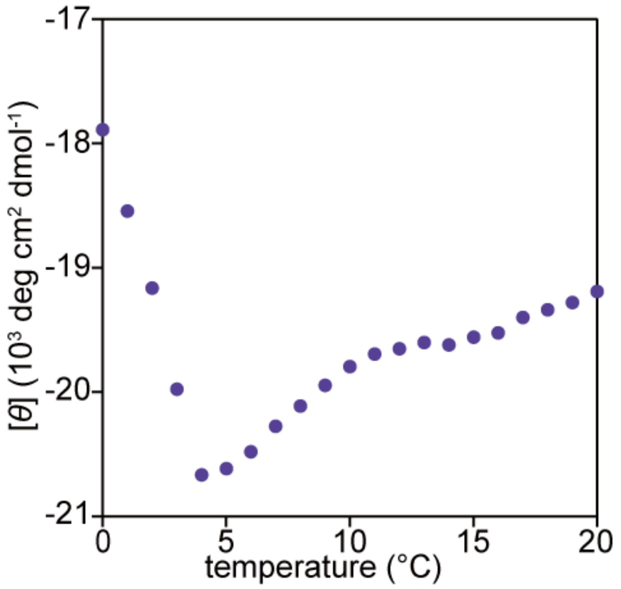

Fig B: Far-UV CD spectra and fluorescence spectra of h-apoMb in 12 mM sodium citrate at 8°C under various conditions. Far-UV CD spectra (A) and fluorescence spectra (B) at pH 6.0 (blue), at pH 6.0 and 8 M urea (red), at pH 4.0 (green), and at pH 2.0 (orange). (A) Circles show the mean residue ellipticity of N_{eq} (blue), M_{eq} (green), and U_{eq} (red) at 222 nm obtained by urea-induced equilibrium unfolding. (B) Fluorescence spectra of N_{eq} (blue), M_{eq} (green) and U_{eq} (red) reproduced by the urea-induced equilibrium unfolding. λ_{max} of each fluorescence spectrum are labeled.

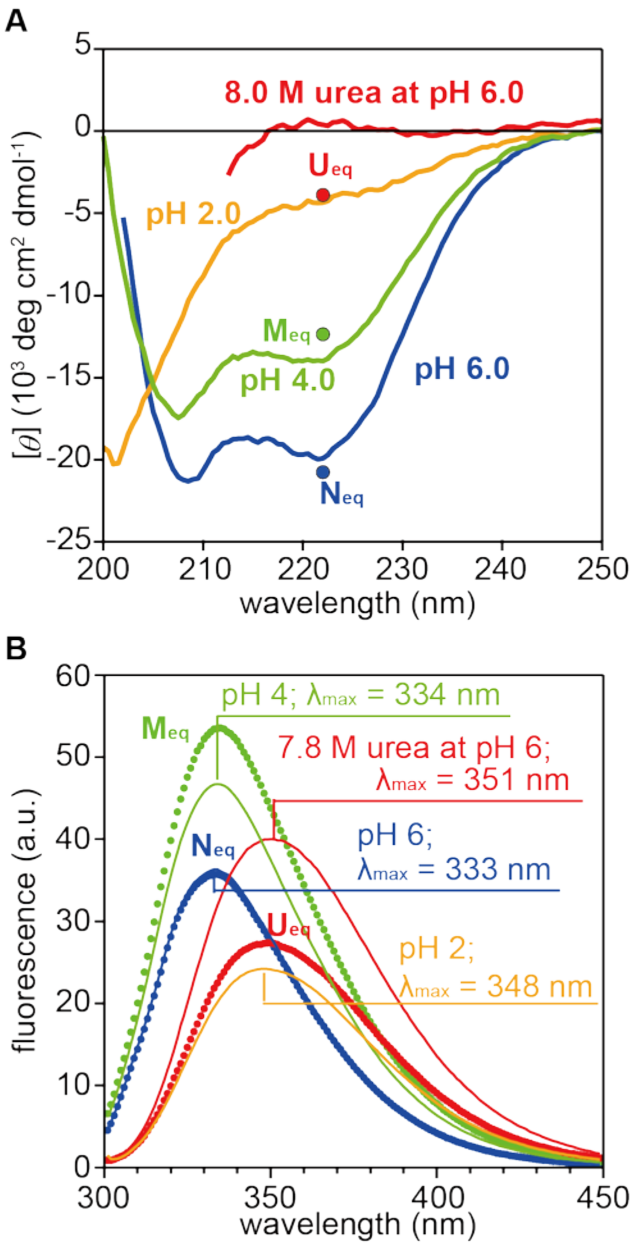

Fig C: Time-dependent changes in fluorescence during the folding of h-apoMb at pH 6.0 and 0.8 M urea compared with those initiated at pH 2.0. The lines in dark colors indicate the reaction initiated at pH 6.0 and 0.8 M urea, while the lines shown by light colors indicate the reaction initiated at pH 2.0. Red, yellow, blue, green, and purple lines represent the reactions measured at 0.13 M, 0.23 M, 0.33 M, 0.43M, and 0.53 M urea, respectively.

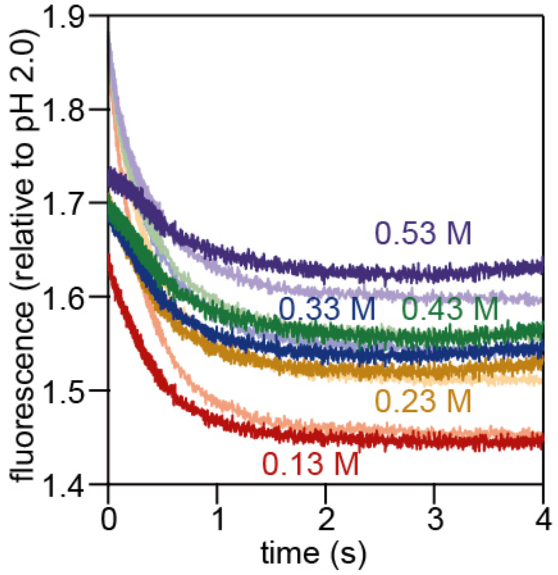

Fig D: urea-dependence of the rate constants and the cumulative amplitudes of refolding and unfolding, and population of each species of h-apoMb calculated by the quantitative modeling assuming four-state schemes. (A) The three four-state kinetic schemes of folding considered here. Panels B, E, and H show chevron plots, kinetic amplitudes, and equilibrium population, respectively, calculated based on Scheme S1. Panels C, F, and I show chevron plots, kinetic amplitudes, and equilibrium population, respectively, calculated based on Scheme S2. Panels D, G, and J show chevron plots, kinetic amplitudes, and equilibrium population, respectively, calculated based on Scheme S3. The circles shown in panels B, C, and D are the rate constants obtained by refolding (green, blue, and cyan) and unfolding (orange and red) experiments using CF- and SF-fluorescence. The black and colored lines shown in panels B, C, and D are apparent rate constants and elementary rate constants, respectively, predicted by the kinetic modeling. The open circles shown in panels E, F, and G are cumulative kinetic amplitudes obtained by refolding (green and blue) and unfolding (orange and red) experiments using CF- and SF-fluorescence, whereas the filled circles shown in blue and red are equilibrium values from the refolding and unfolding experiments, respectively. The black and colored solid lines shown in panels E, F, and G are the equilibrium values and the cumulative amplitudes, respectively. Colored dashed lines show the fluorescence for each state (color codes are shown in the panels). The dotted lines shown in panels H, I, and J represent the equilibrium population of N_{eq} (blue), M_{eq} (green), and U_{eq} (red) states obtained by equilibrium measurements. The solid lines in panels H, I, and J represent the population predicted by the kinetic modeling (color codes are shown in the panels). The regions where the results of kinetic modeling differ from the experimental results are circled.

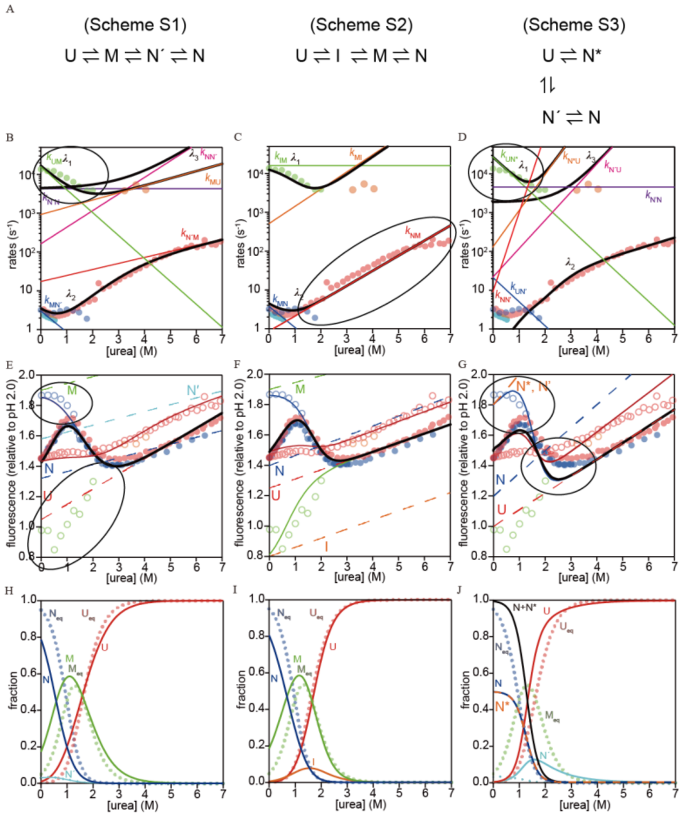

References

1. Chen YH, Yang JT, Chau KH (1974) Determination of the helix and beta form of proteins in aqueous solution by circular dichroism. Biochemistry 13: 3350-3359.

2. Sabelko J, Ervin J, Gruebele M (1998) Cold-denatured ensemble of apomyoglobin: Implications for the early steps of folding. J Phys Chem B 102: 1806-1819.

3. Dasmeh P, Kepp KP (2013) Unfolding simulations of holomyoglobin from four mammals: identification of intermediates and beta-sheet formation from partially unfolded states. PLoS One 8: e80308.

4. Williams RW, Chang A, Juretic D, Loughran S (1987) Secondary structure predictions and medium range interactions. Biochim Biophys Acta 916: 200-204.
